# Active Nematics Reinforce the Ratchet Flow in Dense Environments Without Jamming

**DOI:** 10.1002/advs.202412750

**Published:** 2025-01-23

**Authors:** Yisong Yao, Zihui Zhao, He Li, Yongfeng Zhao, H. P. Zhang, Masaki Sano

**Affiliations:** ^1^ School of Physics and Astronomy Shanghai Jiao Tong University Shanghai 200240 China; ^2^ Institute of Natural Sciences Shanghai Jiao Tong University Shanghai 200240 China; ^3^ Center for Soft Condensed Matter Physics and Interdisciplinary Research and School of Physical Science and Technology Soochow University Suzhou 215006 China; ^4^ Universal Biology Institute The University of Tokyo Bunkyo‐ku Tokyo 113‐0033 Japan

**Keywords:** active nematics, collective motion, controllable cell transport, jamming, ratchet effect

## Abstract

The past decade witnessed a surge in discoveries where biological systems, such as bacteria or living cells, inherently portray active polar or nematic behavior: they prefer to align with each other and form local order during migration. Although the underlying mechanisms remain unclear, utilizing their physical properties to achieve controllable cell‐layer transport will be of fundamental importance. In this study, the ratchet effect is harnessed to control the collective motion of neural progenitor cells (NPCs) in vitro. NPCs travel back‐and‐forth and do not specify head or tail, and therefore regarded as nematics alike liquid crystals. Ratchet and splay‐shaped confinements are crafted to modulate collective cell dynamics in dense environments, while jamming is not explicitly spotted. The adaptation of an agent‐based simulation further revealed how the ratchet's asymmetry and active forces from nematic order synergistically reinforce the directional cell flow. These findings provide insights into topotaxis in cell populations when restricted to crowded 2D ratchets and the mechanisms that regulate collective behavior of the cells.

## Graphical abstract

This study demonstrates enhanced directional migration of neural progenitor cells in micro‐fabricated ratchet geometry, taking advantage of active collective motions. Jamming is eased by cells' spontaneous back‐and‐forth motion as active nematics, answering the long‐standing puzzle of how cells navigate crowded environments over long distances. Agent‐based simulations and active nematic theory provide deeper insights into the mechanisms of collective cell migration.

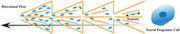

## 1 Introduction

The collective migration and sorting of cells are essential for development, wound healing, and cancer metastasis of organisms.^[^ ^1^, ^2^, ^3^ ^]^ Any mechanism that enables the cells to move towards a predefined location in a dense environment, without resorting to well‐known mechanisms such as chemotaxis or other taxes, is theoretically and practically significant. Cell morphology, phase transitions, cellular forces, geometrical confinement and many other properties have been examined to better understand the migration dynamics within cell layers.^[^ ^4^, ^5^, ^6^, ^7^, ^8^, ^9^, ^10^ ^]^ A recent concept has emerged known as topotaxis^[^ ^11^, ^12^ ^]^ which is different from chemotaxis and other taxes. Little is known regarding how cells conceive and adapt to their physical environment directly. For orientationally ordered systems, boundary conditions can transmit signals of geometrical confinements among cells through their aligning interaction. Thus, topotaxis can occur even through indirect contact considering the existence of local orientational order.

The ratchet effect can be regarded as a vigorous candidate for transporting and separating a large number of mesoscopic or microscopic elements,^[^ ^13^, ^14^, ^15^, ^16^ ^]^ such as colloids, granular particles, molecular motors and so on ^[^ ^17^, ^18^, ^19^, ^20^, ^21^, ^22^, ^23^, ^24^, ^25^ ^]^. Historically, the Brownian ratchet was first proposed as a thought experiment, and later became prestigious with an illustration by Richard Feynman in his thermodynamics lecture.^[^ ^26^, ^27^, ^28^ ^]^ One of the primary mechanisms of the ratchet effect is that in‐between particles are driven back‐and‐forth in an asymmetric potential, resulting in a net motion towards the less demanding direction of the potential as recorded in microfluidic and colloidal systems.^[^ ^29^, ^30^, ^31^, ^32^, ^33^ ^]^ Moreover, in active matter, where particles convert free energy into self‐propelled motion, the ratchet effect has been documented in experiments where bacteria are set in rectified motion in funnel arrays or in ratchet motors.^[^ ^34^, ^35^ ^]^ However, these are wet active systems in which active particles are immersed in a fluid, thus the incompressible condition of the fluid complicates the environment and resulting dynamics. There are hardly any studies on the relationship between orientational order or collective motion and ratcheting, and a lack of experiments or theories describing eukaryotic cells crawling on a substrate. There are studies on spontaneous flows in nematic systems,^[^ ^36^ ^]^ but not incorporating ratchets or other micro‐fabricated structures that can generate controllable directional flow. In this study, we begin with an engineering approach to establish the circumstances under which densely populated neural progenitor cells (NPCs) crawl on a 2D substrate that is surrounded by ratchet ridges fabricated by lithography. These cells align along their elongated direction without distinguishing between heads and tails, exhibiting the so‐called nematic order. If cells align with their heads together, as many other cell types do, it is called polar order. This is a dry active system, and since active stress governs flow and the pattern dynamics, its behavior is expected to be simpler. We explore how nematically ordered cells can migrate in a particular direction depending on physical conditions, such as the surrounding topography and boundary conditions mentioned above. The back‐and‐forth motion of the cells in the ratchet structure creates a directional flow, and when combined with nematic ordering, the flow is significantly enhanced by a splay‐like structure. This is in contrast to polar ordered systems, where it is difficult to change the direction of flow using external topography, and splay may induce a jamming effect. We analyze these phenomena from both experimental and theoretical perspectives, and discuss the future prospects.

## 2 Dynamics on Vast Ratchet Channels

**Figure 1:**
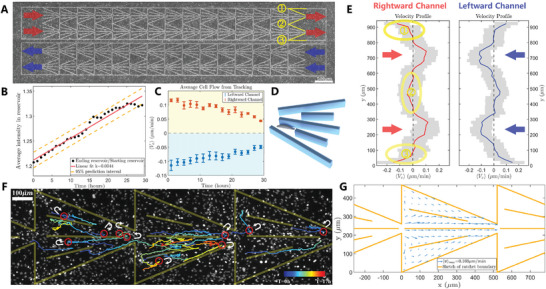
Experimental analysis of collective motion of NPCs on a large‐scale ratchet substrate. A) Bright‐field image of a neural progenitor cell monolayer on a channel pattern consisting of consecutive ratchets (two rightward and two leftward are shown here, which is half of the entire view obtained in one experiment). Red (blue) arrows indicate the designed direction of the channels. Yellow lines show three inversed splay shapes. B) The density (average intensity) ratio between the two rectangular reservoirs connected to either side of the ratchets over time. Data from four channels inside one experimental dish are taken into account. C) The velocity of the cells over time obtained from tracking data of time‐lapse fluorescent images of the nuclei. Each data point is averaged spatially along a channel and then averaged over four identical patterns in one culture dish. D) Illustration of how the NPCs may move and align with ratchet confinement. E) Averaged velocity profile of the *v*_*x*_ component along the y direction. The red curve on the left corresponds to the channel with red arrows in (A) and vice versa. The arrows in (E) point at the inlet (outlet) regions of the channels corresponding to (A). Gray shading indicates the standard deviation. Three ellipses circle the corresponding counterflow region shown in (A). F) Unsmoothed trajectories of different NPCs were obtained by tracking the nuclei (bright white dots) motion along a channel segment. The trajectories are shown with solid lines in which the colors are characterized by time. Light yellow lines sketch the ratchet boundaries. In principle, all cells experience stochastic velocity reversals (see Figure S4; Movie S4, Supporting Information) at an average interval of about two hours (a few typical reversal events are circled out as examples), while cells move towards the smaller gate of the ratchet in the long term. G) Zoomed‐in illustration of the channel's configuration (orange solid lines). Blue arrows plot the average velocity field in a truncated ratchet unit, where the continuous ratchet channels in the experiments are cut into 30 segments, and averaged from 30 h of tracking data inside the segments. Grid size: 35 µm × 35 µm. The walls are double the height of a cell, so that NPCs can hardly cross the boundary. The splay‐shaped angle φ is $\frac{\pi }{4}$.

NPCs naturally exhibit a stochastic back‐and‐forth motion when they are cultured in a dense population on Petri dishes, and collectively manifest active nematic properties. Because of their elongated shape,^[^ ^37^ ^]^ cell bodies align with their neighbors along the long (extended) axis through steric exclusion,^[^ ^38^ ^]^ which results in an ordered state, as in the case of two‐dimensional nematic liquid crystals.^[^ ^39^, ^40^ ^]^ To identify the ratchet effect in a crowded environment, we designed ratchet structures (**Figure** **1**G orange lines) to navigate the direction of cell motion in vitro. A single ratchet segment has a small 50 µm gate and a 470 µm “gate” on the other side, which are bridged successively into large‐scale asymmetric corridors. This induces more challenges when cells attempt to move against the narrow gate. NPCs are seeded on a polydimethylsiloxane (PDMS) substrate, with walls approximately double the height of a cell body (Figure 1D). This hinders individuals from travelling across the ridges. We performed time‐lapse observation under an inverted fluorescent microscope of a near‐confluent dish (Figure 1A shows half area of a common time‐series capture). During observation, there are always cells moving in both directions related to the aligned orientation of the cell. The prior directions of the ratchets are indicated by the red and blue arrows in Figure 1A, which point to the inlet/outlet positions. Four rightward channels accompanied by four leftward channels were evaluated by tracking the movement of cell nuclei labelled by H2B‐mCherry in sequential images. The rectangular reservoirs equipped at both ends of the ratchet show an increase in the density ratio (Figure 1B) indicating that more cells are transported towards the direction pointed by the arrows in Figure 1A. We further analyzed the time evolution of the horizontal velocity component *v*_*x*_ close to the inlet/outlet centers (extending 100 µm up and down from the center of the 50 µm gate), then averaged along the *x* direction for each ratchet (leading to a box size of vertical 200 µm × total channel length), and finally averaged for four different channels with the same geometry (Figure 1C). Compared with the ballistic velocity of the NPCs, which is approximately 0.5 µm *min* ^−1^ (Figure S5, Supporting Information), space‐time averaged coarse grained velocity shows the uni‐directional flow produced by the ratchet with a magnitude of about 0.1 µm *min* ^−1^, and the separation (gray dashed line in Figure 1C) between the leftward (negative) and rightward (positive) flow is apparent. Over time, the average flow intensity decreased, which was probably the result of cumulative phototoxicity. The average velocity field inside the ratchet segments is overlapped in Figure 1G with blue arrows, pointing consistently towards the narrower side. Figure 1E shows the velocity profile *v*_*x*_(*y*) along the *y* direction from the two enclosed boxes in Figure 1A. Both were averaged in time and *x* direction. The position of the arrows in Figure 1E correspond with the position of the arrows in Figure 1A with the same color as in the *y* direction. Opposite flows emerged at the upper and lower boundaries and central zone (marked with orange ellipses in Figure 1E) because of the backward splay‐like structures (three yellow splays in Figure 1A), which are described in the next section.

Tracking results of NPCs in these ratchets also confirm the co‐existence of velocity reversals and persistent ratchet movement based on the detailed trajectories (Figure 1F), with a reversal time of approximately one to two hours (Figure S4, Supporting Information) inside the ratchets. This nematic behavior was further verified by the local orientation order (Figure S2A, Supporting Information) calculated by the structure tensor method (see Section 5 and Method). In the bright‐field images, the cell bodies are neatly aligned with the PDMS ridges to form a typical nematic order.

## 3 Splay Confinement with Reservoirs

**Figure 2:**
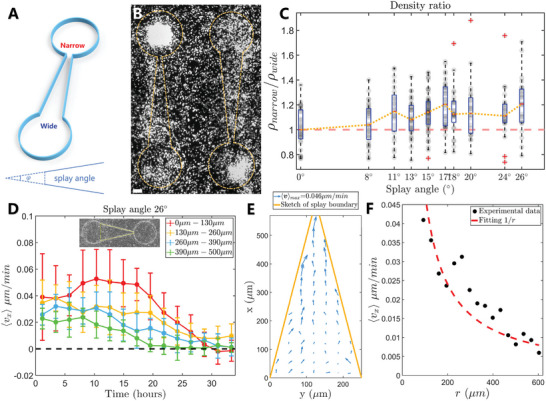
Splay‐shaped structures detached from consecutive ratchets for a more detailed study. A) An illustration of a splay‐shaped pattern with reservoirs at both ends for analyzing the density difference. B) Fluorescent image of cell nuclei distribution after 24 h of observation. Orange dashed lines show the pattern configuration. Scale bar, 50 µm. C) Density (indicated by the fluorescent intensity) ratio of the circular reservoirs connected to the narrow/wide end of the splay varying the splay angle. This boxplot combines the results of four experiments (dishes) 30 h after confluence, and the gray scatters show the entire data. The pink dashed line indicates the reference line of the identical density, and the orange dotted line connects the medium value given by the boxplot. Sample size *N* = 32 for the first 5 angles, and *N* = 24 for the last five. D) Velocity component *v*_*x*_ evolution at 4 different splay segments along the *x* direction (yellow splay of the inset, averaged vertically and over 12 identical patterns in one experiment) over time. 0 µm means exactly at the narrow end of the splay. Error bars indicate the standard error of the mean. E) Velocity field inside the splay (wall indicated by the orange lines) averaged over 12 patterns and 30 h. Grid size: 35 µm × 35 µm. F) Average of the velocity field along the radial direction in (E) and fits the data with $\frac{1}{r}$ where *r* is the distance from the intersection point of two orange lines.

To determine the influence of splay frameworks on yielding steady cell flow in ratchets, we designed analogous configurations (**Figure** **2**A) to enclose NPCs. The ridges were still printed high enough to impede crossing events as the ratchet predecessors. Two reservoirs were attached to both ends of each splay aisle to evaluate the extent of transport. A typical final stage from the fluorescent image of cell nuclei (Figure 2B) indicated an accumulated state at the reservoirs adjacent to the narrow gates (upper‐left and lower‐right in Figure 2B), whereas the opposite remains relatively vacant, which can be visualized dynamically in Movie S2 (Supporting Information). We determined the dependence on the splay angle by varying the gate width of the splay patterns, and averaged the multiple patterns with the same angle for each data point. The boxplot for the density ratios (Figure 2C) was calculated by comparing the average pixel intensity values between the twin reservoirs, which showed linearity with the number of cells (Figure S1, Supporting Information). The ratio increased concomitantly as the angle increased, whereas saturation likely occurred because of the finite reservoir size, as the velocity component (directional flow) evolution decays over time (Figure 2D). A positive *v*_*x*_ indicated a rightward flow to the narrow gate of the splay and the inset shows the orientation. We also analyzed the averaged velocity field (Figure 2E) from 30 h of tracking data with 12 identical patterns in one experiment. The arrows dominantly pointed toward the narrow gate and the flow flourished as it got closer to the singular aster defect center (Figure 2F), which is discussed in Section 5. To characterize how the nematic interaction participates in directional transport inside the ratchets, we broke up the whole into parts using simulation trials.

## 4 Agent‐Based Model of Dry Active Nematics

To corroborate this collective transport effect for nematic systems, we tailored an active nematic model^[^ ^41^ ^]^ for particle simulation in 2*D*. Self‐propelled particles displaced at a constant velocity magnitude *v*_0_ for computational efficiency, following the direction of a unit vector $\vec{e}$ controlled by the orientation θ of the particle. This discrete time model takes three factors into account: alignment, repulsion, and angular noise within a certain interaction radius *r*_0_, based on the following formula:

(1) $${\vec{r_{i}}}^{t+1}={\vec{r_{i}}}^{t}+v_{0}\vec{e}\left(\theta_{i}^{t+1}\right)$$

(2) $$\theta_{i}^{t+1}=arg\left\{\varepsilon_{t}{\left\langle sgn\left[\cos(\theta_{i}^{t}-\theta_{j}^{t})\right]\vec{e}\left(\theta_{j}^{t}\right)\right\rangle }_{j}+\beta {\left\langle {\vec{r_{ji}}}^{t}\right\rangle }_{j}\right\}+\eta \chi_{i}^{t}$$

The interaction range between particles was selected as 1. For the first alignment term, and for each particle *i*, there is a chance of *a* ∈ [0, 0.5] at each step that the orientation flips 180° (ϵ_*t*_ changes from +1 to −1) resulting in a reversed motion as a nematic (or pure polar, if *a* appears to be zero) system. In the pairwise repulsion, ${\vec{r_{ji}}}^{t}$ indicates the unit vector which points from another particle *j* to particle *i* at time *t*, which is manipulated by a factor β. For the last term, $\chi_{i}^{t}\in \left[-\pi /2,\pi /2\right]$ defines the angular noise from a homogeneous distribution, and η controls its strength. And 〈 〉_*j*_ stands for the average with respect to *j* particles.

**Figure 3:**
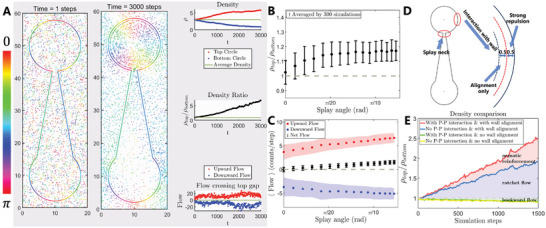
Characterization of the ratchet effect with nematic interaction in silico. A) Snapshot of particle simulation at step 1 and step 3000. The color of the particles indicates the nematic orientation. Top right panel: time series of densities in top (red) and bottom (blue) reservoirs. Average density (green). Middle right: density ratio (black) between top and bottom reservoirs. ρ_*top*_/ρ_*bottom*_ = 1 (green). Bottom right: flow crossing top (red) and bottom (blue) gaps. zero flow (green). 〈ρ〉 = 3, *v*_0_ = 0.18, β = 0.3. B) Density ratio between the two reservoirs as a function of the splay angle. C) Flow at the splay neck as a function of the splay angle, the shaded region and error bars show the standard deviation. D) Demonstration of the simulation boundary and the particle‐wall interaction. E) Density ratio over time calculated by dividing the number of particles in the two circular reservoirs. Red (blue) line plots the density ratio evolution in a simulation with (without) the particle–particle interaction, and green (yellow) lines simulate situations where the boundaries do not align particles (repel particles only). B and C: error bar indicates standard deviation from 300 simulation trials, see supplementary text in detail. Unit of flow: number of particles per step.

**Figure** **3**A shows a simulation snapshot, and the color of the particles is displayed according to their current nematic orientation. Stationary particles are situated as boundaries that mimic splay ridges with reservoirs. These particles do not enroll in updates, while furnishing enormous repulsion when other mobile particles intrude into certain territory. This provides aligning guidance at a moderate distance (Figure 3D). The density evolution, the corresponding density ratio, and the upward/downward flow at the splay neck were recorded together. We altered the splay angle via tuning gate width to resemble the experiments in the previous section. The accumulation ratio and particle flow exhibit a monotonic increase (Figure 3B,C). The plots were acquired by averaging 300 simulation trials (see Materials and Methods). After 500 simulation steps, the density ratio (Figure 3B) was saturated slightly as the angle increased, which was likely due to limited size of reservoirs and possible effects by gentle jamming or diffusion (Figure S7, Supporting Information), and is consistent with the appearance of the experiments (Figure 2C). Figure 3C shows that the downward flow always exists and causes fluctuation, whereas the upward flow is elevated with the splay angle.

The parameters were selected from a homogeneous nematic state and the directional transport persists and extends over a wide range from our parameter settings, *v*_0_ and β (Figure S8A, Supporting Information). Although it is not possible to fully relate all the parameters from the experiments to a relatively simplified simulation model, we can still gain insight from a few comparisons. If 500 simulation steps encompass a 30 h experiment (because the density ratio resembles each other in Figure 2C and Figure 3B), one step corresponds to $\frac{30\;\;\mathrm{hours}}{500}∼3.6\;\;\mathrm{mins}$. The velocity reversal time is approximately 1 h in the experiments (Figure S4, Supporting Information), so $a∼\frac{3.6\;\;\mathrm{mins}}{60\;\;\mathrm{mins}}=0.06$ in the simulation. This suggests that a one‐day observation represents the early stage in the simulation in Figure 3A, whereas the motility of the cells decreases due to phototoxicity (Figure S5B, Supporting Information), which adds difficulty to an even longer observation. The length scale was designed to be 1 unit: 10 µm where the reservoir radius (channel length) are 10(30) units in simulation and 100(300) µm in the experiments. Thus, we can evaluate $v_{0}∼\frac{0.5\;\mu m\;\min^{-1}}{10\;\mu m\;{\mathrm{unit}}^{-1}}\times 3.6\;\;\min\;{\mathrm{step}}^{-1}=0.18\;\;\mathrm{unit}\;{\mathrm{step}}^{-1}$ in the simulation.

To determine whether the directed flow solely stemmed from the ratchet asymmetry of the substrate potential, we performed simulations with identical initial and boundary conditions, while eliminating the particle–particle (P–P) interaction. The accumulation ratio with the P–P interaction prevailed over its rival (the one without nematic interaction) in Figure 3E, whereas eliminating the wall‐particle alignment resulted in a slight counter flow, which may be bounced back by the immense wall repulsion at the splay neck. As *v*_0_ is augmented, the density ratio grows accordingly (Figure S8, Supporting Information). Therefore, the effect from a collective point of view highlighting active nematic interactions within the splay boundary cannot be ignored. In the next section, we analyze how this nematic interaction at a high density contributes to reinforced transport inside the ratchets.

## 5 Nematic Order Toward Reinforced Directional Transport

At a single particle level without interaction, various types of ratchets consisting of a Brownian particle in anisotropic periodic structures, driven by time‐periodic forcing^[^ ^42^, ^43^, ^44^ ^]^ have been proposed. Directional current **J** was calculated in the framework of stochastic dynamics. When a particle experiences stochastic back‐and‐forth motion in anisotropic periodic structures, a similar current **J** contributes to the ratchet flow (Figure 3E), and is reinforced by the nematic interaction as follows.

For quantitative analysis, splay patterns can be regarded as part of a +1 topological defect (aster defect).^[^ ^45^, ^46^ ^]^ Consider a 2D director field **n** = (cos θ, sin θ), where the orientation angle writes θ = *k*ϕ + θ_0_ = ϕ for a aster defect (*k* = 1, θ_0_ = 0). Integer topological defects or naturally formed ±1/2 defects have already been discovered that have a profound impact on the dynamics of living cells.^[^ ^47^, ^48^, ^49^ ^]^ The traceless nematic order tensor is defined as $Q=2S\;\;(\mathrm{nn}-\frac{1}{2}I)$,^[^ ^40^, ^50^ ^]^ where *I* represents the identity matrix. The scalar order parameter *S* has a maximum value of one, which indicates perfect order, and a minimum of zero, which indicates that no alignment exists. Setting the order parameter **Q** traceless in active nematics allows for a concise representation of the orientational order that excludes isotropic contributions, and provides a clear measure of the anisotropy in the system. Then, the 2 × 2 tensor is: $Q=S\left(r\right)\left(\begin{array}{cc} \cos2\phi & \sin2\phi \\ \sin2\phi & -\cos2\phi \end{array}\right)$.

Without curvatures, the linear terms can qualitatively represent the dynamics of the system. Then the simple active force can be described by **f** ^*a*^ = −ζ∇ · **Q**.^[^ ^39^, ^51^ ^]^ Finally, we arrive at:

(3) $$f^{a}=-\zeta \left[S^{\prime }\left(r\right)+2\frac{S(r)}{r}\right]e_{r}$$

ζ is usually regarded as the extensile parameter, which is used in active matter systems to describe the nature of constituent particles and whether they experience pushing or pulling forces from their neighbors or the substrate. Adherent NPCs are considered to be extensile from the correlation of density‐alignment fluctuations.^[^ ^39^, ^52^ ^]^ They are prone to elongate and form nematic order, yielding a positive ζ value. The unit vector **e**_*r*_ points away from the presumed +1 aster defect center. For splay configuration, *S*(*r*) can be considered constant (cells tactfully align with the boundary by measurement, Figure S2, Supporting Information), assuming its derivative *S*′(*r*) to be zero inside the channel. As a result, the active force may be simplified as: $f^{a}\approx -\zeta \frac{2S}{r}e_{r}$. Then, the flux through the splay pattern can be evaluated as $J=\rho v=-2\rho \zeta S\frac{e_{r}}{\gamma r}$, where velocity **v** is derived from γ*v* = **f** ^*a*^ in an overdamped approximation. The $v∼\frac{1}{r}$ relationship is examined through experimental data in Figure 2F. γ stands for the friction coefficient. This flux originates independently from a usual ratchet contribution considering asymmetry between the two tails, but from the standpoint of nematic interaction with local order among the cell layer. This is inherent to many ratchet structures that are overlooked easily. As cell division naturally proceeds, one can expect the flux to be greater at a higher density over time during the growth and development of the cell layer with the existence of splay‐shaped ratchets and nematic interaction. Integration results in the total flux in a defined splay as follows:

(4) $$I=\int_{\phi_{1}}^{\phi_{2}}J\cdot nd\phi =-2\rho \zeta S\frac{|\phi_{2}-\phi_{1}|}{\gamma }$$

where φ = |ϕ_2_ − ϕ_1_| is the opening angle of the splay pattern, in accordance with the monotonic increase of flow with the splay angle described beforehand. A negative sign indicates that the direction of the total flux is opposite to **e**_*r*_, pointing from the wide gate towards the narrow gate (Figure S2B, Supporting Information). However, when the variation of the scalar order parameter is not negligible, *S*′(*r*) emerges very close to the splay neck (Figure S2C, Supporting Information) because of a distorted orientation, which suggests a more considerable active flow. A similar simulation result on density dependence (Figure S8B, Supporting Information) also displayed a linear relationship as predicted in the equation. Overall, this flux amplifies ratchet flow to promote more efficient transport in a dense environment where robust nematic interaction exists.

## 6 Conclusion and Discussion

Our study explored the realization of double‐sided ratchets to control the collective migration of NPCs in vitro. We observed significant flows in the directional migration of cells when they were cultured on vast ratchet channels. Specifically, they exhibited a reinforced preferential movement towards the direction of the active force in a crowded environment where they elongated and formed a splay‐shaped nematic order, as evidenced by quantitative analysis in experiments and particle simulations. This phenomenon was consistently observed across experimental replicates and was validated with the particle model simulation and active nematic theory. Evidence of targeted cell flow was provided by the velocity field either inside the splay (Figure 2E) or ratchet segments (Figure 1G). This negates the possibility of different cell‐division rates between circular confinements. At the early stage of the splay experiments (far before saturation), the relative cell increment rate of the intensity inside the reservoirs connected to the narrow gate increased monotonically with the splay angle (Figure S3F, Supporting Information), and that connected to the wide gate fluctuated around the natural cell‐division rate. This is only possible if we consider the transported cells as dominant contributors; otherwise, the growth rate inside the circular confinement near the wide gate will also increase.

Several limitations should be considered when interpreting the results. Our experiments were conducted in vitro and may not fully represent the complexity that occurs in vivo. Besides, NPCs have difficulty surviving at a dilute density and from frequent stimulation by lasers. Therefore, we imitated low‐density behavior with simulation by killing the interaction terms or by setting a small ρ value, which may have limitations.

An in vivo phenomenon which might be related to our work is the rostral migratory stream (RMS). The RMS is a long, specialized migration pathway in the brains of certain animals, where neuronal precursors originating from the subventricular zone (SVZ) travel to the main olfactory bulb (OB). Cells in the RMS are believed to move by chain migration, while the detailed mechanism still remains debating. The motion of the cells in the RMS were examined to be bi‐polar,^[^ ^53^ ^]^ similar to our observation of back‐and‐forth motion of neural progenitor cells in the experiments. Consecutive ratchet channels in our setup could probably create chain migration representing the thin and long pattern discovered in the brain, which are able to generate directional flows at high density as the RMS.

NPCs' response to the ratchet effect provides deep insight into the role of topotaxis underlying cell migration, and offers comprehension into the principles governing spontaneous order formation, and the emergence of collective motion in soft matter systems. By further inducing curvatures instead of straight walls or building defect‐like dislocations^[^ ^54^, ^55^ ^]^ in the ratchets to create more substantial active flux, more significant directed transport could be generated with a similar ratchet size for nematic systems. Moreover, the possibility of regulating cell dynamics steadily (Figure S6, Supporting Information) holds promise for developing novel strategies in regenerative medicine, facilitating targeted cell transport, and developing cell‐based therapies for neurological disorders and brain injuries.

## 7 Experimental Section

### Cell Culture

Neural progenitor cells of mice were kindly given away from Ryoichiro Kageyama, and mutants labelled by H2B‐mChrry were made by Kyogo Kawaguchi at Kageyama's laboratory. Cells are cultured mainly following the protocols in ref. [39]. DMEM/F‐12, HEPES, no phenol red (Thermo Fisher, 11039021) was used as medium base supplemented with FGF‐basic (Wako, 060‐04543), EGF (Thermo Fisher, 53003‐018), and N‐2 Max (R&D Systems, AR009). The 35*mm* plastic dishes (Falcon, 353001) or glass‐bottom dishes (Nunc, 150682) were pre‐coated with 10 × diluted Matrigel (Corning, 356231) before seeding the cells overnight, then removed the coating agent and placed NPCs with 2*mL* medium. The cultures were incubated at 37°*C* with 5% carbon dioxide at saturated humidity, and we changed 2*mL* medium every 2 days. During passage, StemPro Accutase (Gibco, A1110501) was used to disassociate cells from the substrate, and Stem‐Cellbanker (ZENOAQ, 11922) was applied for making frozen stocks.

### Substrate Preparation

The patterns were designed in AutoCAD and produced chromium mask accordingly, then employed SU8 3025 (Kayaku) for lithography at 1000 *rpm* for generating ∼20 µm ridges on silicon wafers. The polydimethylsiloxane (PDMS) substrate was then molded regarding,^[^ ^56^, ^57^ ^]^ mixed with curing agent at 7: 1, then poured on the silicon wafer and heated at 80°*C* for 2 h on the hot plate in a clean bench. After solidification, plasma cleaner was applied to improve hydrophilicity of the surfaces, and finally sterilized with 75% ethanol for 30 min before Matrigel coating.

### Imaging Method

NPC cell culture on glass‐bottom dish with PDMS substrate was grown in incubator before confluent, then observed through bright field (BF) and fluorescence on Leica DMi8 upright microscope. Tokaihit incubator, which can maintain temperature and humidity and carbon dioxide concentration, is assembled on the microscope for long‐term observation of live cells, usually lasted 24–36 h for a single experiment. Dual images were taken every 6 min to reduce phototoxicity. For Figure S3 (Supporting Information), photos are taken every 10 min in order to maintain a steady growth shortly after passage, but they are not suitable for tracking.

### Data Analysis

The tracking of cells was carried out through *TrackMate* plugin^[^ ^58^, ^59^ ^]^ in ImageJ^[^ ^60^ ^]^ with *Cellpose* detector (finding the nuclei at each frame)^[^ ^61^ ^]^ and simple LAP algorithm. *Cellpose* detector in *TrackMate* is able to identify irregular elliptical shapes. The velocity data obtained from tracking was smoothed using moving‐average of 5 points. In the verification of linear relationship between the number of cells and the average pixel intensity, cells were counted in *Ilastic* ^[^ ^62^ ^]^ with machine learning method. Before obtaining the pixel intensity values, the images were calibrated by the following:

(5) $$\mathrm{Calibration}=\frac{\mathrm{Original}\;\mathrm{image}-\mathrm{dark}\;\mathrm{current}\;\mathrm{image}}{\mathrm{Fluorescence}-\mathrm{dark}\;\mathrm{current}\;\mathrm{image}}$$

where dark current signals were subtracted, and divided by an empty fluorescence image (without any sample) to further calibrate the possible inhomogeneity of the incoming light source.

2*D* orientation field was calculated by the BF images with the structure tensor method: $G=\left(\begin{array}{cc} I_{x}^{2} & I_{x}I_{y}\\ I_{y}I_{x} & I_{y}^{2} \end{array}\right)$, where *I*_*x*_ and *I*_*y*_ stand for the pixel intensity gradient of a given image *I*. After smoothing with Gaussian filter and removing edges, the local orientation angle can be defined by: $\theta =\frac{1}{2}\arctan\left(\frac{G_{xy}+G_{yx}}{G_{xx}-G_{yy}}\right)$.

### Autocorrelation Calculation from Tracking

To estimate the reversal time of NPCs, we select relatively long trajectories (more than 5 h). From tracking data, the displacement $d_{i}\left(t\right)$ and location $r_{i}\left(t\right)$ were obtained of the *i* ^*th*^ cell at time *t*. The orientation θ_*i*_(*t*) of cells can be estimated through taking the arc‐tangent of *y* and *x* component from the displacement.

In a time lapse observation, the cosine autocorrelation function (*ACF*) which stands for cells' velocity direction can be calculated as:

(6) $$\begin{array}{cc} ACF_{direction}\left(\tau \right) & =\langle \cos\left[\theta_{i}\left(t+\tau \right)-\theta_{i}\left(t\right)\right]\rangle \\ & =\frac{1}{N}\sum_{i}\cos\left[\theta_{i}\left(t+\tau \right)-\theta_{i}\left(t\right)\right] \end{array}$$

where *N* is the number of total selected tracks. On the other hand, the cosine autocorrelation function for nematic orientation can be written as:

(7) $$\begin{array}{cc} ACF_{nematic}\left(\tau \right) & =\langle \cos2\left[\theta_{i}\left(t+\tau \right)-\theta_{i}\left(t\right)\right]\\ & =\frac{1}{N}\sum_{i}\cos2\left[\theta_{i}\left(t+\tau \right)-\theta_{i}\left(t\right)\right] \end{array}$$

The autocorrelation function of direction was fitted with an exponential function $Ae^{-2\tau /\tau_{c}}$, where *A* is a coefficient (set to be 1 since *ACF* = 1 when τ = 0) and τ_*c*_ is the critical time scale for velocity reversal (Figure S4, Supporting Information).

### Growth Rate Calculation

The cell layer grows exponentially as cell division occurs over time *t*: *N*(*t*) = *N*_0_ *e* ^α*t*^, where *N*_0_ is the initial cell number when observation started, and constant τ implies how fast the cells can divide on average. At early stages, the exponential function can be further approximated by Taylor expansion:

(8) $$\begin{array}{cc} N(t) & =N_{0}\left[1+\left(\alpha t\right)+{\left(\alpha t\right)}^{2}+\cdots \right]\\ & \approx N_{0}+N_{0}\alpha t. \end{array}$$

If we fit the evolution of cells by a linear function *f*(*t*) = *b* + *kt* (Figure S3, Supporting Information), the growth rate can be obtained as α = *k*/*b*. The doubling time *T*_*d*_, which determines how long it takes for the cells to divide into twice of the initial population, can also be estimated by setting $2N_{0}=N_{0}e^{\alpha T_{d}}$, giving rise to $T_{d}=\frac{\ln2}{\alpha }$. Cells undergoing time lapse observation grow slightly slower compared with those inside typical incubators.

### Details for Particle Simulation

Parameters for Figure 3 and Movie S3 (Supporting Information): *L*_*x*_ = 20, *L*_*y*_ = 60, *a* = 0.06, *step* = 3000 (Figure 3A; Movie S3, Supporting Information), *step* = 500 (Figure 3B,C), *step* = 1500 (Figure 3E), ρ = 3, η = 0.15, *v*_0_ = 0.18, β = 0.3, splay angle φ = 20°.

Parameters for Figure S6 (Supporting Information): *L*_*x*_ = 50, *L*_*y*_ = 100, *a* = 0.05, *step* = 100000, ρ = 2, η = 0.12, *v*_0_ = 0.2, β = 0.4, φ = 20°.

Parameters for Figure S7 (Supporting Information): *L*_*x*_ = 20, *L*_*y*_ = 60, *a* = 0.06, *step* = 1500, ρ = 3, η = 0.15, *v*_0_ = 0.18, β = 0.3.

Parameters for Figure S8 (Supporting Information): *L*_*x*_ = 20, *L*_*y*_ = 60, *a* = 0.06, *step* = 1000, ρ = 3, η = 0.15, φ = 18°.

Parameters for Figure S8B (Supporting Information): *L*_*x*_ = 20, *L*_*y*_ = 60, *a* = 0.06, *step* = 500, η = 0.15, *v*_0_ = 0.18, β = 0.3, φ = 18°.

Conditions for Figure 3B,C and Figure S8B (Supporting Information): Each point is averaged by restarting the simulation for 300 times shuffling the seed of random number generator. And at each run, stop at 500 simulation steps to collect the results. Error bar shows standard deviation over the 300 attempts.

## Conflict of Interest

The authors declare no conflict of interest.

## Supporting information

Supporting Information

Supplemental Movie 1

Supplemental Movie 2

Supplemental Movie 3

Supplemental Movie 4

## Data Availability

The data that support the findings of this study are available from the corresponding author upon reasonable request.
