# Supplementary material for: Active Nematics Reinforce the Ratchet Flow in Dense Environments Without Jamming
Source: Adv Sci (Weinh). 2025 Jan 23;12(11):2412750. doi: 10.1002/advs.202412750 (PMC11923915; doi:10.1002/advs.202412750)
Supplement: Supplementary file 1 — Supporting Information [file ADVS-12-2412750-s005.pdf]

ADVANCED SCIENCE
Open Access

Supporting Information

for *Adv. Sci.*, DOI 10.1002/advs.202412750

Active Nematics Reinforce the Ratchet Flow in Dense Environments Without Jamming

*Yisong Yao, Zihui Zhao, He Li, Yongfeng Zhao, H. P. Zhang and Masaki Sano**

Supporting Information for

Active Nematics Reinforce the Ratchet Flow in Dense Environments without Jamming

Yisong Yao, Zihui Zhao, He Li, Yongfeng Zhao, Hepeng Zhang, Masaki Sano*

*Corresponding author: sano.masaki@sjtu.edu.cn

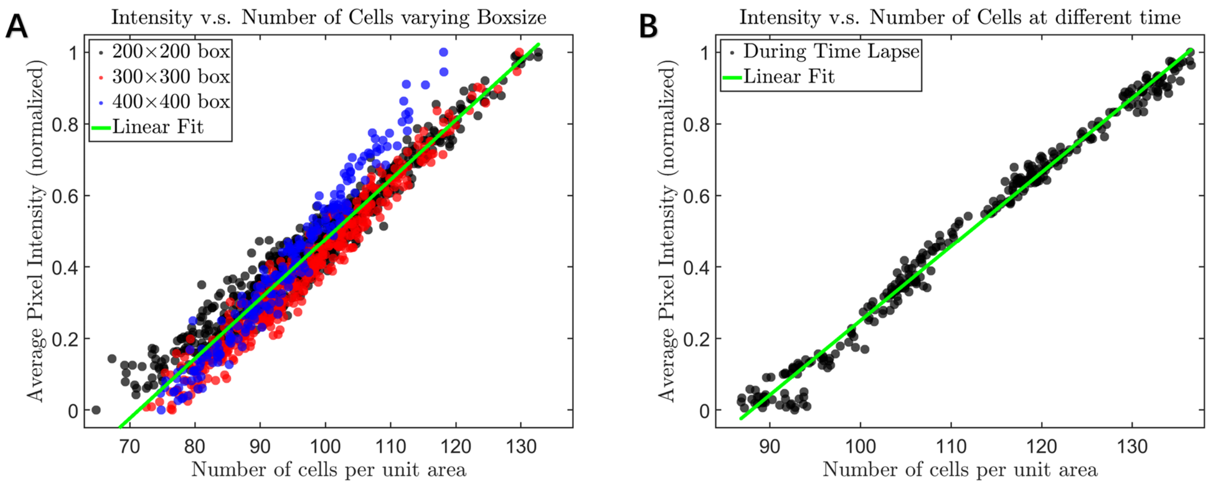

Figure S1

Relation between cell numbers and the fluorescent intensity of images. A In an experimental observation, several fluorescent frames (of the nuclei) were selected and cropped into rectangular pieces at different box sizes (200, 300 and 400 pixels). The average intensity (by ImageJ) and the number of cells (by Ilastic) are measured within each piece. **B** At a fixed $200\ pixel \times 200\ pixel$ box, the average intensity and the number of cells are measured during time lapse. **A, B** Green line shows the linear fitting of the data. Unit area means a $100\ pixel \times 100\ pixel$ square box.

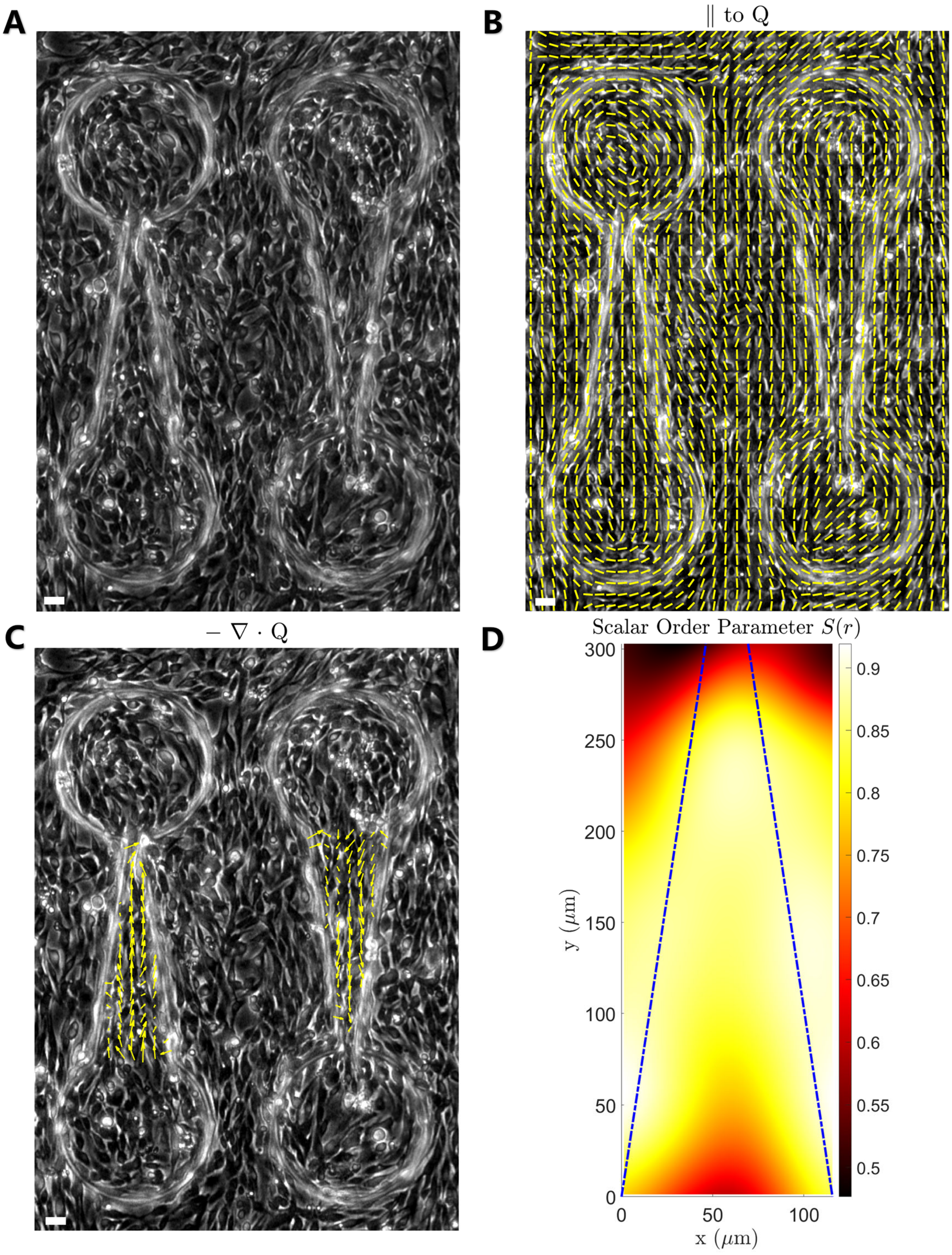

Figure S2

Active force and scalar order parameter distribution of neural progenitor cells with splay boundary condition. A The bright field image of the splay pattern with NPCs. **B** Principal direction of the nematic order tensor $\boldsymbol{Q}$ (yellow short lines) overlapped on a phase contrast image. Averaged for 100 frames with box size of 32 μm, and shifting the grid at half box size (staggered grid) for better spatial resolution. **C** Vector field of $-\nabla \cdot \boldsymbol{Q}$ (yellow arrows) obtained from B close to the splay region which defines the direction of active force with a positive

extensile parameter ζ. **D** Distribution of the scalar order parameter $S(r)$ inside an upward splay closure obtained from the left part of B, and blue dashed lines specify the boundary of splay confinement. A sharp drop of $S(r)$ close to splay neck originates from the connection to the circular reservoir causing a rapid change in orientation. Scale bar, 30 μm.

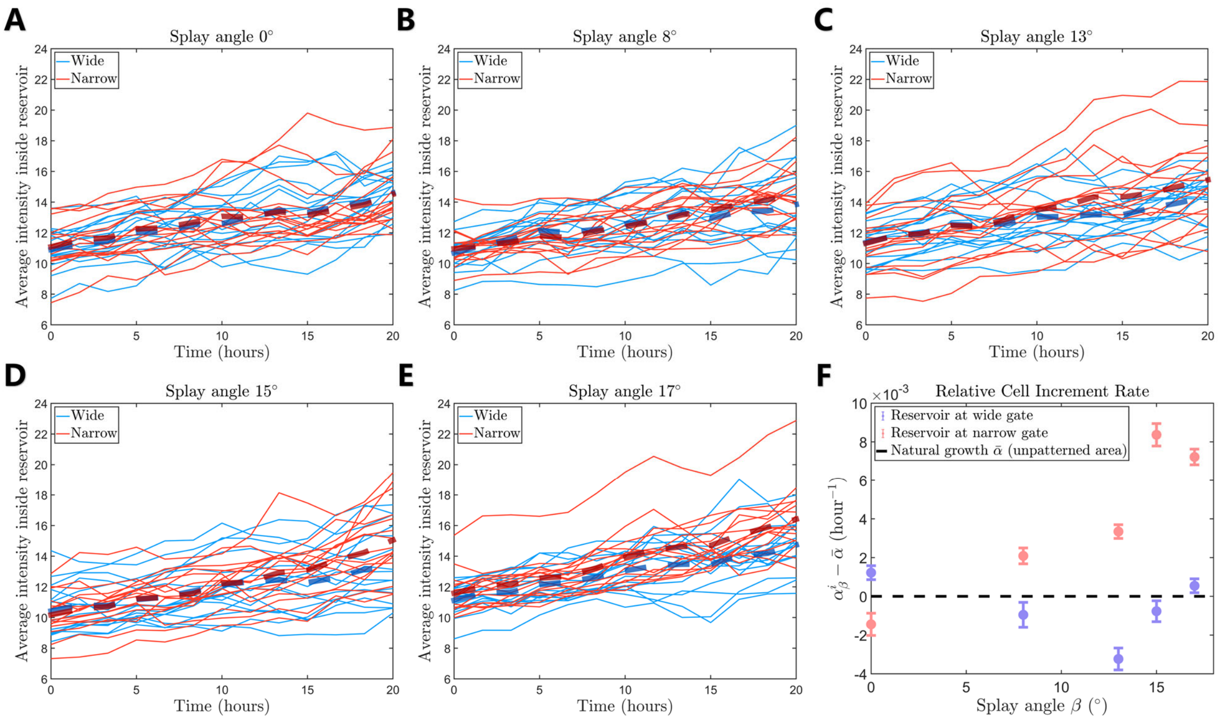

Figure S3

Time evolution of the intensity (number of cells) in a splay experiment. A-E The evolution of average intensity in the circular reservoirs at early stages (5 hours after passaging to the PDMS pattern) to get rid of saturation in the reservoirs. Each blue (red) thin line demonstrates the average intensity inside a single reservoir connected to the wide (narrow) end of splay, while the thick dashed blue (red) line plots the mean value of the blue (red) thin lines. Linear fitting of each dashed line defines the increase rate α_β^i inside the reservoirs connected to the splays at each angle β, where i denotes wide (blue) or narrow (red). **F** Slopes derived from linear fitting of the thick lines in A-E, and error bars show the 95 % confident intervals from the fitting. Difference between the averaged increase rate for reservoirs with wide (narrow) gate α_β^i and the mean proliferation rate $\bar{\alpha}$, i.e. $\alpha_\beta^i - \bar{\alpha}$, is plotted with blue (red) symbols as a function of β. $\bar{\alpha}$ is the fitted result from the evolution of average intensity on remaining unpatterned area in the same experiment as the estimate of mean proliferation rate of cell number.

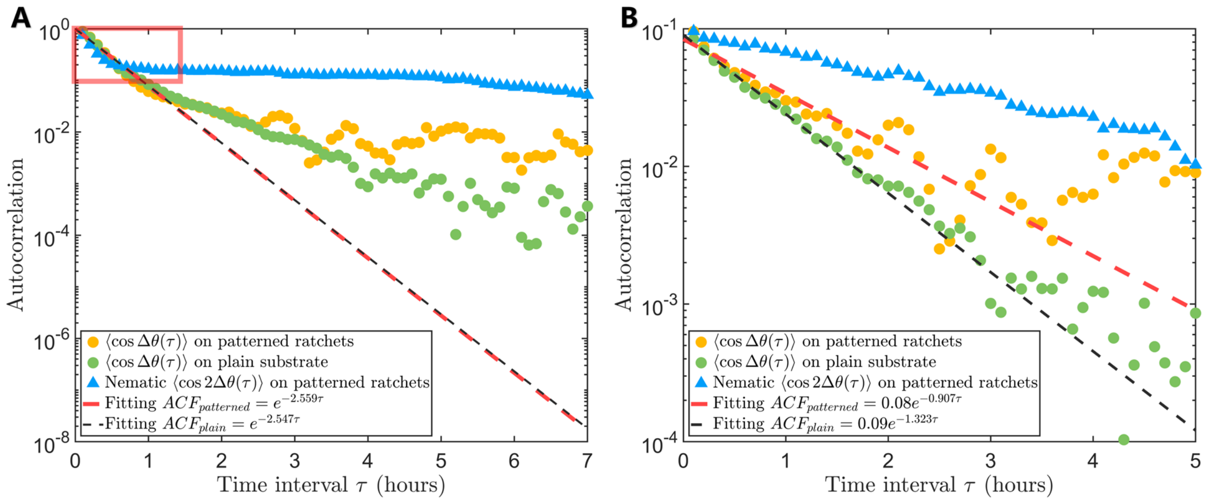

Figure S4

Autocorrelation function of NPC's orientation from tracking trajectories. A The autocorrelation function of cell's moving direction $\langle \cos \Delta\theta(\tau) \rangle$ and nematic orientation $\langle \cos 2\Delta\theta(\tau) \rangle$ calculated from 20,324 (72,117) selected trajectories that last over 50 frames (longer than 5 hours) on a ratchet-patterned (plain) area. There are two slopes in the semi-log plot. The initial fast decay gives a reversal time about 47 mins. These fast decays may be caused by jiggling of cells during cell division, or possibly wrong tracking results. **B** By removing the initial fast decays, we obtain slower decays in the autocorrelation function. Fitting this result with $\exp(-2\tau/\tau_c)$ gives the estimation of velocity reversal time $\tau_c \sim 2$ hours, which is more consistent to reference [39].

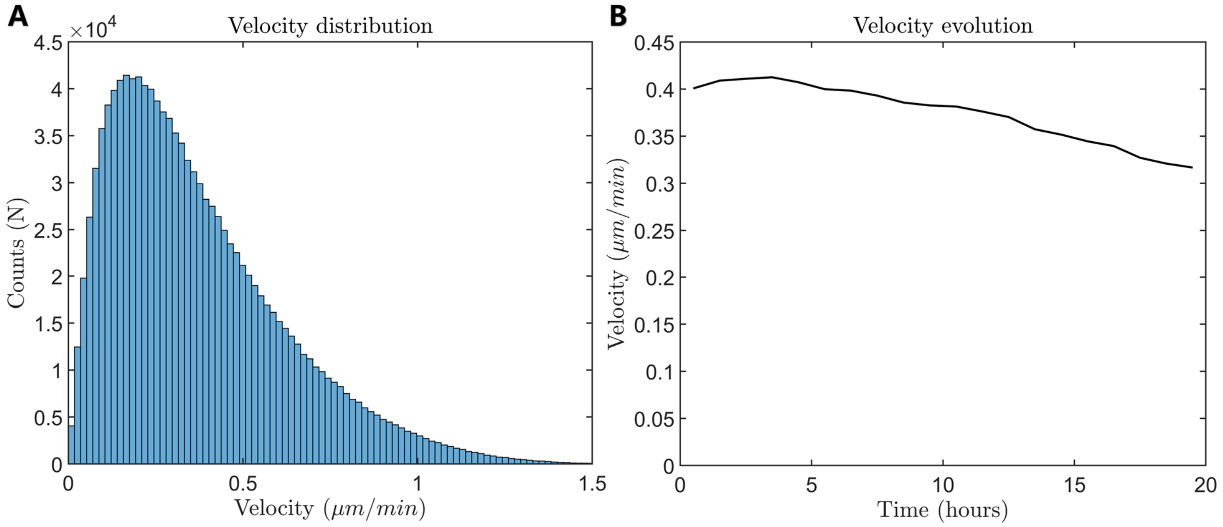

Figure S5

Properties of the cells' motility on the planar substrate of glass-bottom dishes. A Histogram of instantaneous velocity of the cells. A total of 54,368 tracked trajectories are taken into account in a 20 hours' time lapse observation, and the distribution of NPCs' ballistic velocity is plotted. **B** The velocity data is averaged every 1 hour, and tends to decrease over time due to phototoxicity.

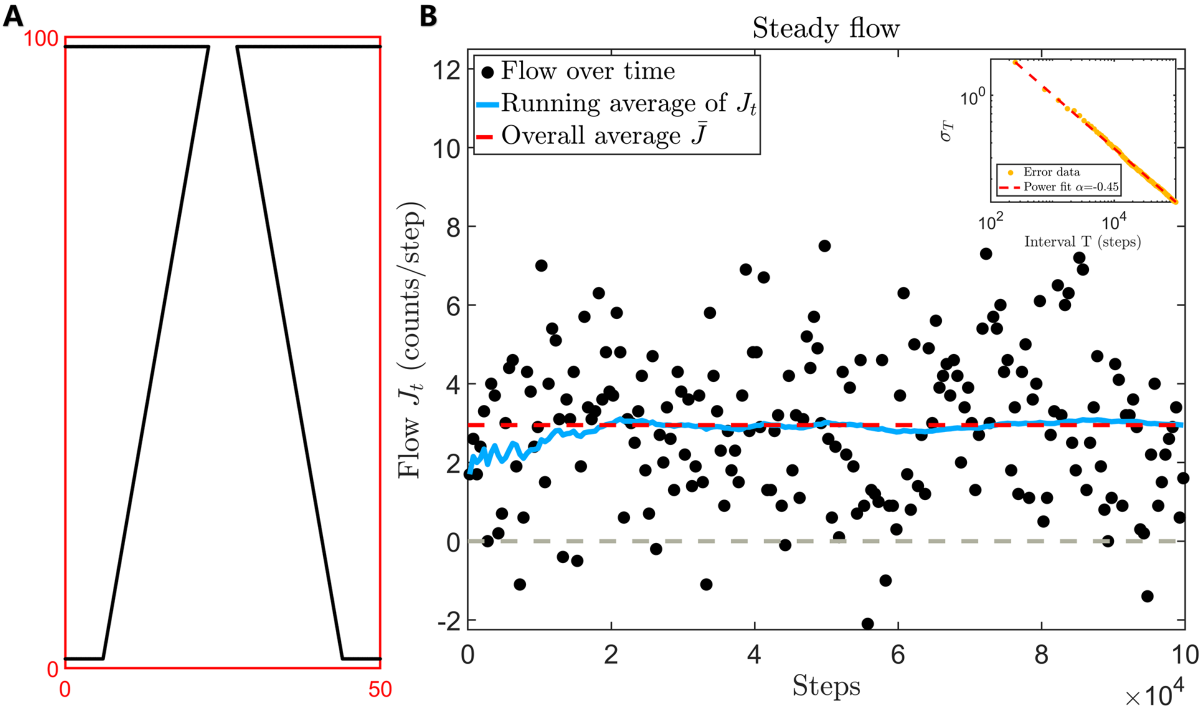

Figure S6

Simulating steady flow with periodic boundary. A Illustration of the simulation box to produce steady flow. Red lines indicate periodic boundaries (top to bottom, left to right), and black lines shows solid boundary whose interaction is kept the same as Fig. 4D. **B** Net flow (upward flow minus downward flow) over time obtained from the configuration in A at the splay neck. Positive value means the flow is upward. The red dashed line plots the average of all the points $\bar{J}$. Blue line shows the average flow from beginning ($t = 0$) till the current step which converges according to the central limit theorem. Each black dot is averaged for 500 steps in the simulation. Inset: standard error $\sigma_T = \sqrt{\frac{\sigma^2}{T}}$ of the flow J with the mean value of $\bar{J}$ over the time interval T is compared with a power fit $\sim T^{-1/2}$. Standard deviation $\sigma = \sqrt{\frac{1}{T}\sum_{t=1}^{T}(J_t - \bar{J})^2}$.

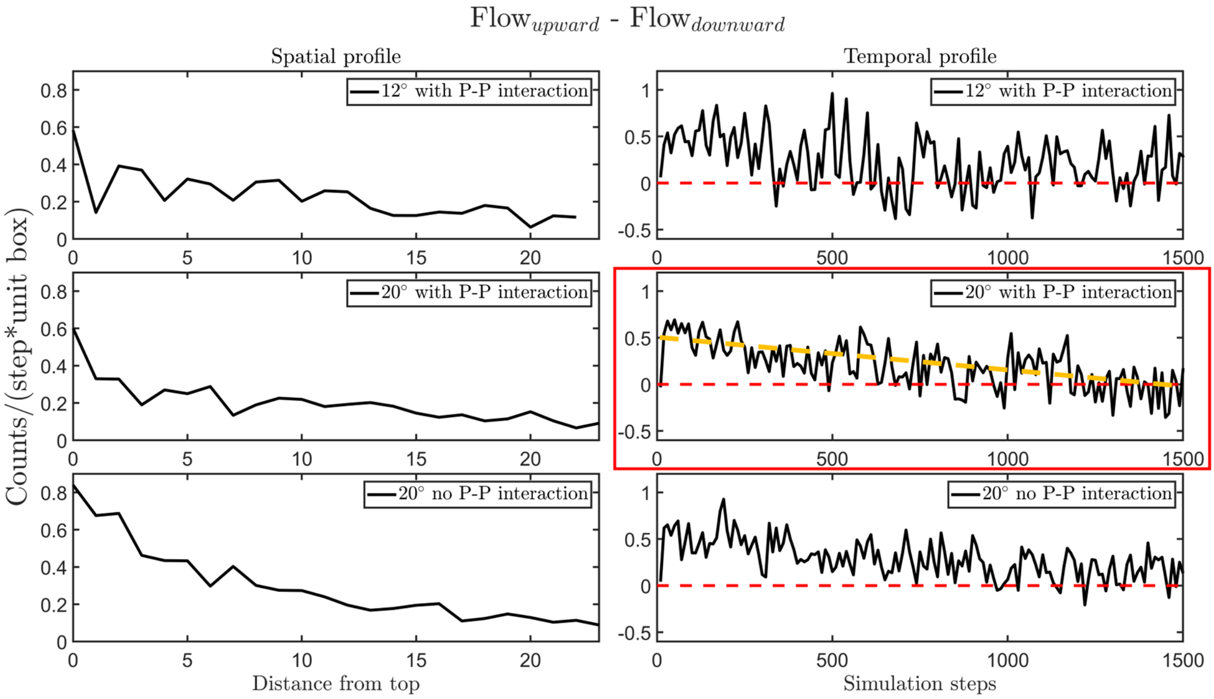

Figure S7

Spatial and temporal profile of net flow inside a simulated splay pattern. Spatial (left column) and temporal (right column) profile from three different configurations: a smaller splay angle with particle-particle interaction (first row), a larger angle with (second row) and without (third row) interaction. The red box shows evidence of slight jamming/diffusion where the net flow decays fastest among the three. Without interaction (bottom right), the flow also decreases due to a limited number of total particles, but it decreases more slowly than the one with interaction.

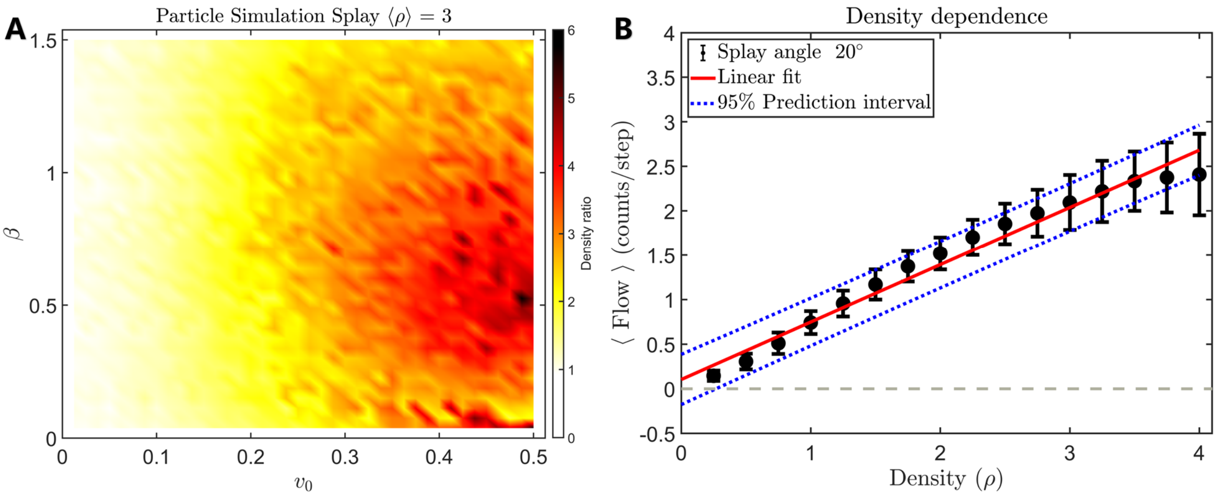

Figure S8

Change in parameters influences the flow strength but does not necessarily reverse the direction. **A** Phase diagram of density ratio between reservoirs varying velocity and repulsion. In the simulation of splay patterns, we compare the ratio of number densities between two reservoirs after 1,000 steps. Almost the entire parameter space demonstrates accumulation at the reservoir close to the smaller opening of splay patterns. **B** Particle simulation of flow dependence on density. The number of particles moving upward minus the number of particles moving downward at splay neck per step varying the overall density (number of particles in a 1 × 1 box) of simulation system. Averaged by restarting the simulation for 300 times varying the seed of random number generator, and stop at 500 simulation steps. Error bar shows standard deviation.

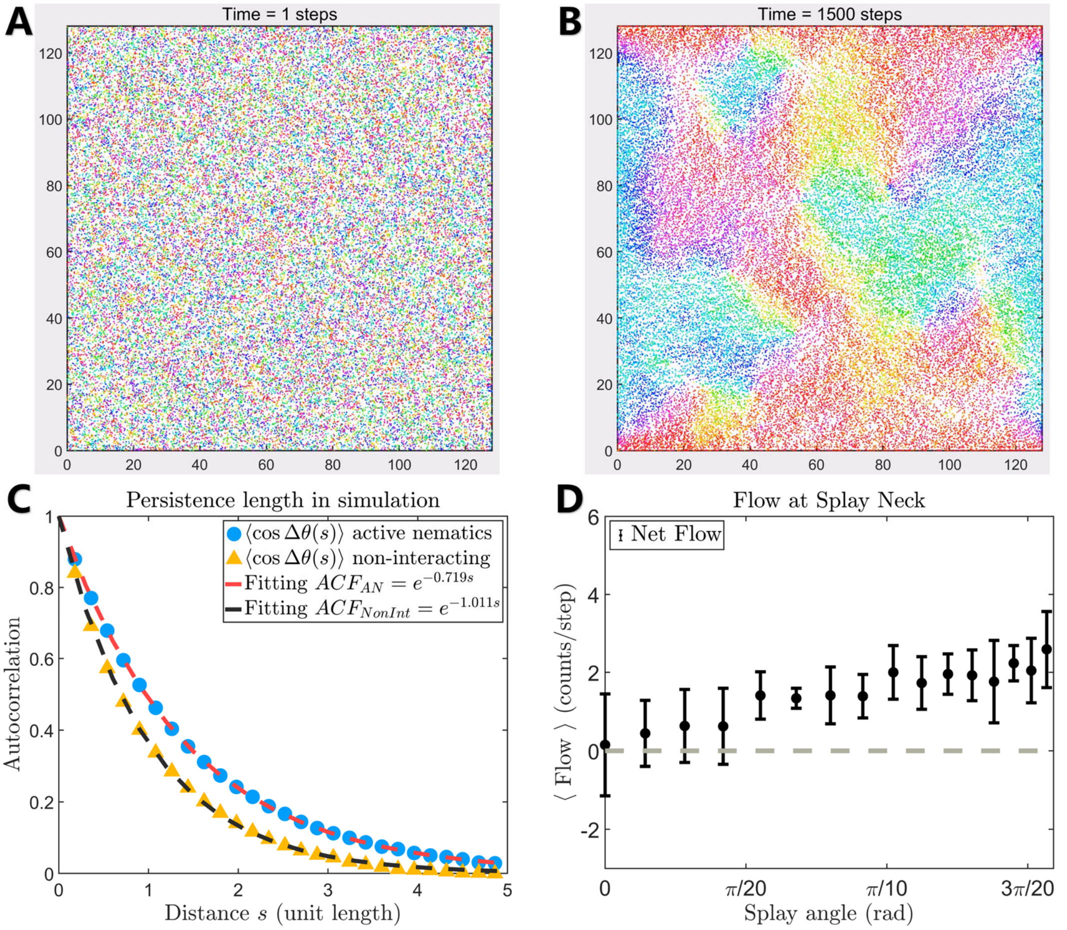

Figure S9

Persistence length of the particles in the simulation. A, B Particle simulation in a box size of 128×128 with periodic boundary condition without any confinement. Colors are indicated by the nematic orientation the same as Figure 3A in the manuscript, and other parameters also remain the same as Figure 3A. **C** The autocorrelation function $\langle \cos \Delta\theta(s) \rangle$ (blue circles, starting from the time point in B where a steady nematic state is reached) along each particle's distance s of the trajectory contour, and averaged for $N = L_x \times L_y \times \rho = 128 \times 128 \times 3 = 49152$ particles. The persistence length can be estimated by fitting $\langle \cos \Delta\theta(s) \rangle = e^{-\frac{s}{L_p}}$. Thus, $L_p = \frac{1}{0.719} \approx 1.39$. By killing the interactions (orange triangles), the persistence length becomes smaller: $L_p' = \frac{1}{1.011} \approx 0.99$. **D** Simulation result of directional flow at the splay neck by tripling the length scale (from box size of 20×60 to 60×180) of confinement while maintaining the other parameters within the first 500 steps, and the gap size of the narrow gate becomes 5.84 which is much larger than L_p. Error bar shows the standard deviation from 10 simulations replicas with each splay angle varying random seeds.

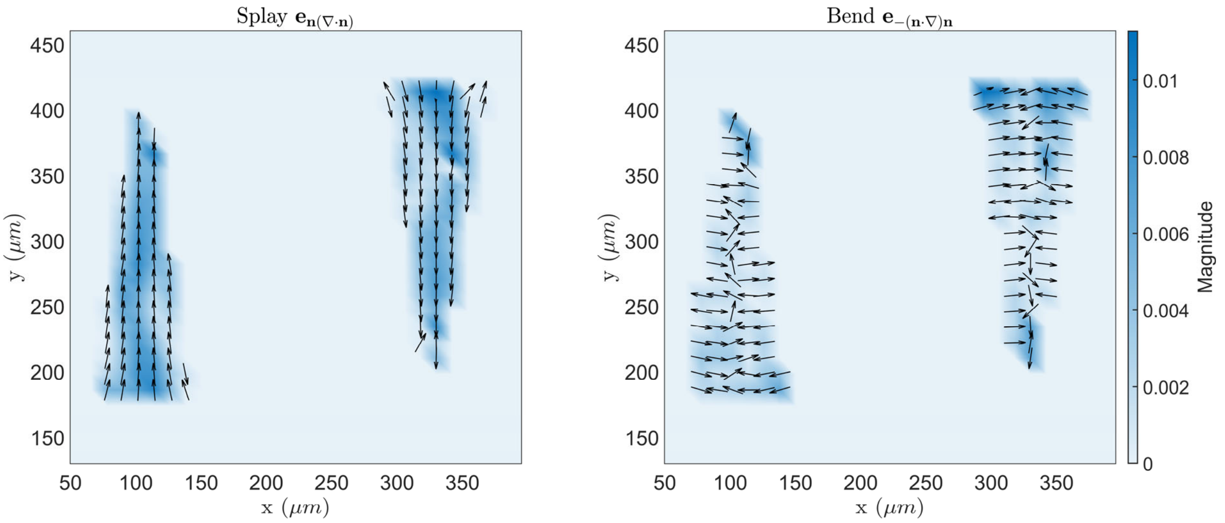

Figure S10

Comparison between splay and bend mode from experimental data inside the splay patterns. Deformations of the two-dimensional nematic field can be decomposed into bend mode $-(n \cdot \nabla)n$ and splay mode $n(\nabla \cdot n)$ where n represents the director field. We calculated two modes from the same experimental data as in Figure S2. The bend mode has much less contributions to the directional flow, mainly pointing to the circular direction which are perpendicular to the walls (boundary), thus not contributing to the directional flow along the ratchet. The magnitude (colormap) is also smaller compared with the splay mode.

Movie S1.
Experimental bright field movie of NPC's motion in a ratchet channel.

Movie S2.
Experimental fluorescent movie of NPC's motion under splay confinement.

Movie S3.
Particle simulation movie with the same configuration of splay experiment by a dense and dry active nematic model.

Movie S4.
Fluorescent live images of NPCs labelled by H2B-mcherry with tracked trajectories and spots.
